# Nociceptive pain unmasking a serious pathology – paraneoplastic hypertrophic osteoarthropathy: A case report

**DOI:** 10.1097/MD.0000000000015900

**Published:** 2019-06-07

**Authors:** Athena Cristina Ribigan, Ovidiu Lucian Bajenaru, Florina Anca Antochi, Ovidiu Alexandru Bajenaru

**Affiliations:** a University Emergency Hospital Bucharest, Neurology Department; b Ana Aslan National Institute of Geriatry and Geriatrics, Geriatry and Geriatrics Department; c University of Medicine and Pharmacy Carol Davila Bucharest, Department of Clinical Neurosciences, Bucharest, Romania.

**Keywords:** hypertrophic osteoarthropathy, paraneoplastic, Pierre Marie–Bamberger syndrome

## Abstract

**Rationale:**

Hypertrophic osteoarthropathy, also named Pierre Marie–Bamberger syndrome, represents a rare medical condition that may be considered either a primary or a secondary disease, and lung malignancies are responsible for more than two-thirds of the cases with secondary forms of the disease.

**Patient concerns:**

We present the case of a 41-year-old man referred to our Neurology Department for pain that was considered secondary to cervical disc protrusions. The neurologic examination was normal. However, the general examination showed digital clubbing, right lateral cervical adenopathy, and pachydermia. The radiographic examinations of the upper and lower limbs depicted osseous abnormalities typical for periostosis, and the computed tomography of the thorax showed the presence of a mass lesion in the right upper pulmonary lobe. High values of vascular endothelial growth factor were also found. The patient was admitted to the Pneumology Clinic, where biopsy was performed from the lateral cervical adenopathy.

**Diagnoses:**

The anatomopathological examination revealed multiple neoplastic infiltrates suggestive of adenocarcinoma metastasis. Based on the clinical examination and radiological and histologic findings, the diagnosis of pulmonary adenocarcinoma with lymph nodes metastases and paraneoplastic hypertrophic osteoarthropathy was established.

**Interventions:**

The patient received treatment with nonsteroidal antiinflammatory drugs and opiate analgesics that relieved the pain.

**Outcomes:**

The patient was referred to the Oncology Department for further treatment of the primary pathology. He received different types of chemotherapeutics, immunotherapy, and radiotherapy. However, despite all therapeutic measures, the disease rapidly progressed and the patient died 9 months later.

**Lessons:**

This is an interesting case of a patient with an overlooked pathology, which was refereed to our clinic for further investigations of a pain that was considered neuropathic, secondary to small cervical protrusions. Conversely, the pain proved to be nociceptive and Pierre Marie–Bamberger syndrome was the positive diagnosis in our patient, as it can be associated with numerous diseases, especially of neoplastic origin.

## 1 Introduction

Hypertrophic osteoarthropathy, also named Pierre Marie–Bamberger syndrome, was described at the end of the 19th century and represents a medical condition that may be considered either a primary hereditary disease (very rare) or secondary to other diseases (95%–97% of cases).^[1]^ This syndrome has a few distinct clinical features: periostosis (affecting initially the distal parts of the limbs), digital clubbing, arthralgia, and arthritis, sometimes accompanied by synovial effusion.^[2]^

Secondary forms are due to a wide range of diseases, the most frequently by paraneoplastic syndromes that are associated to lung cancer. However, other nonneoplastic diseases of the lungs can be mentioned, such as infections, inflammatory diseases (sarcoidosis), and arteriovenous malformations, and disease affecting other organs, such as cyanotic heart disease, cirrhosis, and inflammatory bowel disease.^[3]^ Lung malignancies, either primary or metastatic, are responsible for approximately 80% of the cases with secondary hypertrophic osteoarthropathy, and most of the patients are diagnosed with nonsmall-cell lung tumors like squamous cell or adenocarcinoma.^[4]^

The aim of this article is to present the case of a young patient who was referred to a Neurology Clinic for further investigations of a severe chronic pain that was proved to be secondary to osseous abnormalities in the clinical context of a hypertrophic osteoarthropathy. To establish if hypertrophic osteoarthropathy was a primary process or secondary to other disease, different laboratory tests were performed. In conclusion, it was certified that Pierre Marie–Bamberger syndrome had a paraneoplastic origin, as the patient was diagnosed with lung malignancy.

## 2 Case report

A 41-year-old man presented to our Neurology Department with pain at the level of the right shoulder and right interscapular–vertebral region, with onset for about 6 months. In the last 30 days, the pain exacerbated and radiated in the anteromedial part of the arm and clavicular area. The patient also reported pain in the joints of the distal part of upper and lower limbs, especially in the small joints, which afterward became swollen.

The patient presented with a medical history of arterial hypertension for the last 10 years, treated with beta-blockers and sartans, and he was a heavy smoker (in the last period he used only electronic cigarettes).

**Figure 1:**
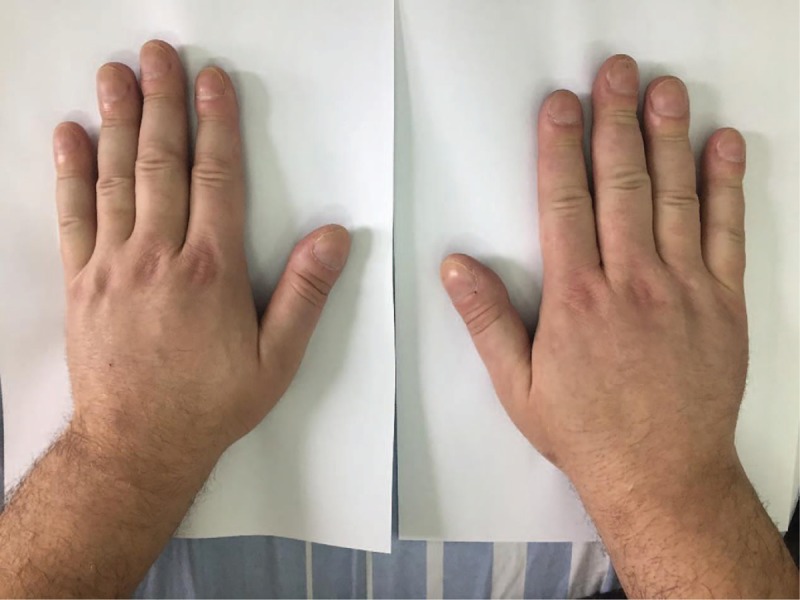
Clubbing of the fingers.

**Figure 2:**
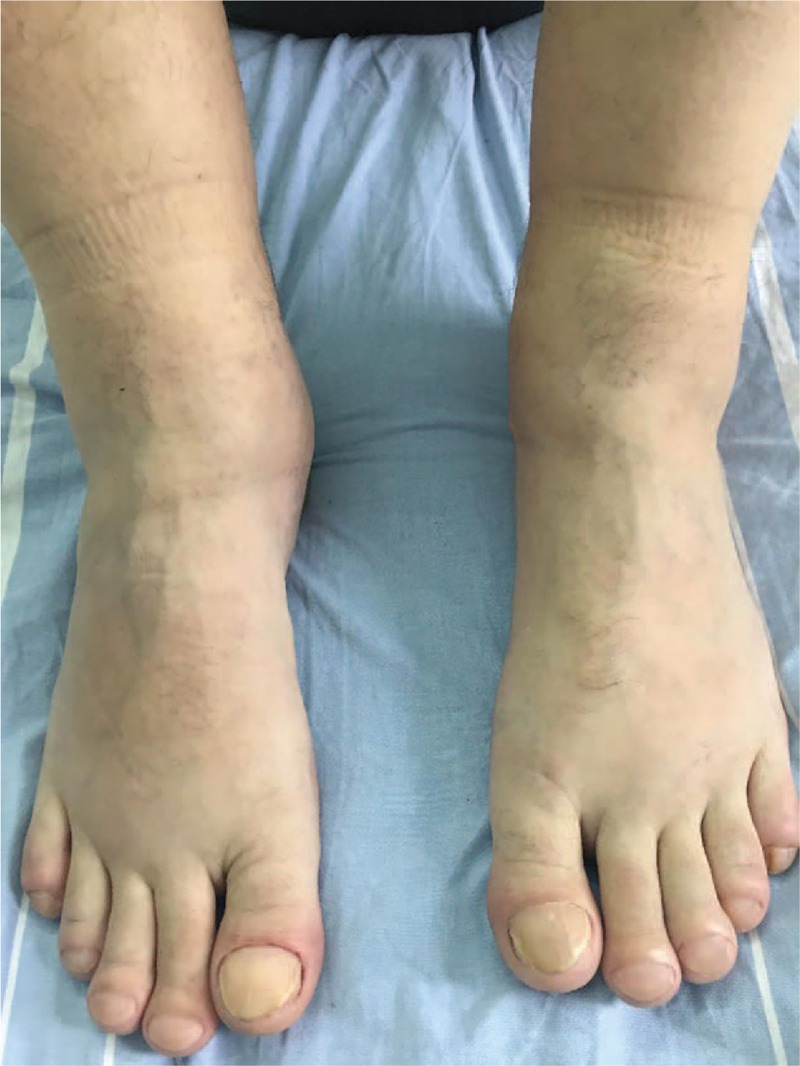
Clubbing of the toes and enlargement of the large joints, swollen extremities with a tubular appearance.

The neurologic examination was normal, except for slightly diminished deep tendon reflexes of the lower limbs. The patient reported pain that did not correspond to any radicular or nerve territory, which was exacerbated by pressure on the distal third of radius and ulna, but was not related to active or passive movements of the cervical spine. The general examination showed clubbing of the fingers and toes (not mentioned initially by the patient, but present for about 1 year) with an increase of the shoe size of more than 1.5 sizes, enlargement of the large joints, swollen extremities with a tubular appearance (Figs. 1 and 2), a right lateral cervical mobile, painless adenopathy of 1 cm diameter, and pachydermia with thickening of the skin of the scalp, forehead, and fingers, with cranial skin folds.

Cervical magnetic resonance imaging performed in another clinic before the admission to our department showed the presence of disc protrusions from C2 to C7, with concomitant C2, C4, and C5 root compressions. The electrophysiological study revealed an active denervation in the territory of the right C5, C6, and C7 roots.

**Figure 3:**
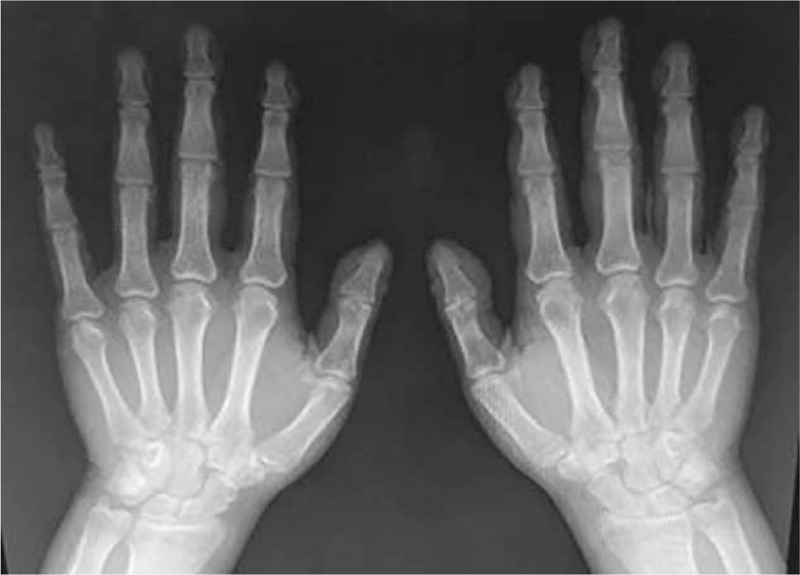
Radiographic examination of the upper limbs.

**Figure 4:**
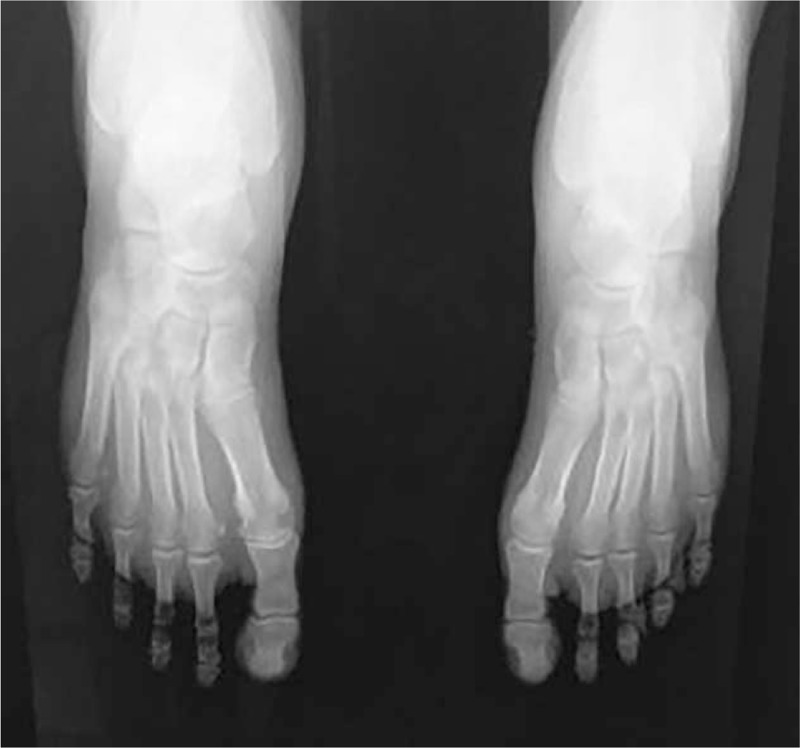
Radiographic examination of the lower limbs.

The radiographic examinations of the upper and lower limbs depicted symmetric osseous abnormalities, typical for periostosis, and linear halving of the diaphysis with an increase in the bone circumference. There were no fractures or cortical destruction (Figs. 3 and 4). Transthoracic echocardiography and electrocardiography were normal.

**Figure 5:**
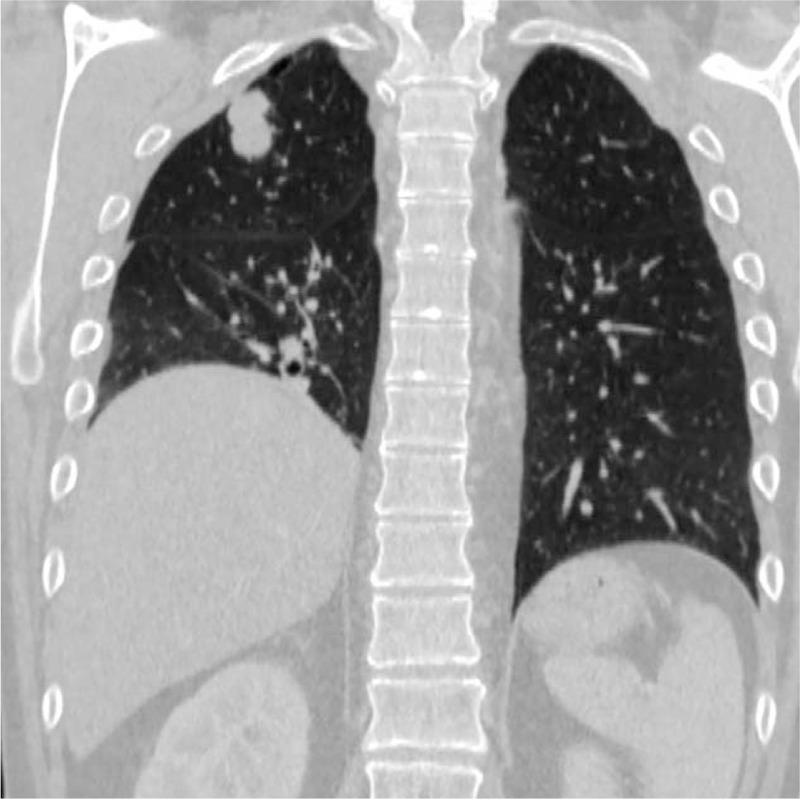
CT scan of the thorax showing the presence of a mass lesion located in the dorsal segment of the right upper pulmonary lobe – coronal view. CT = computed tomography.

**Figure 6:**
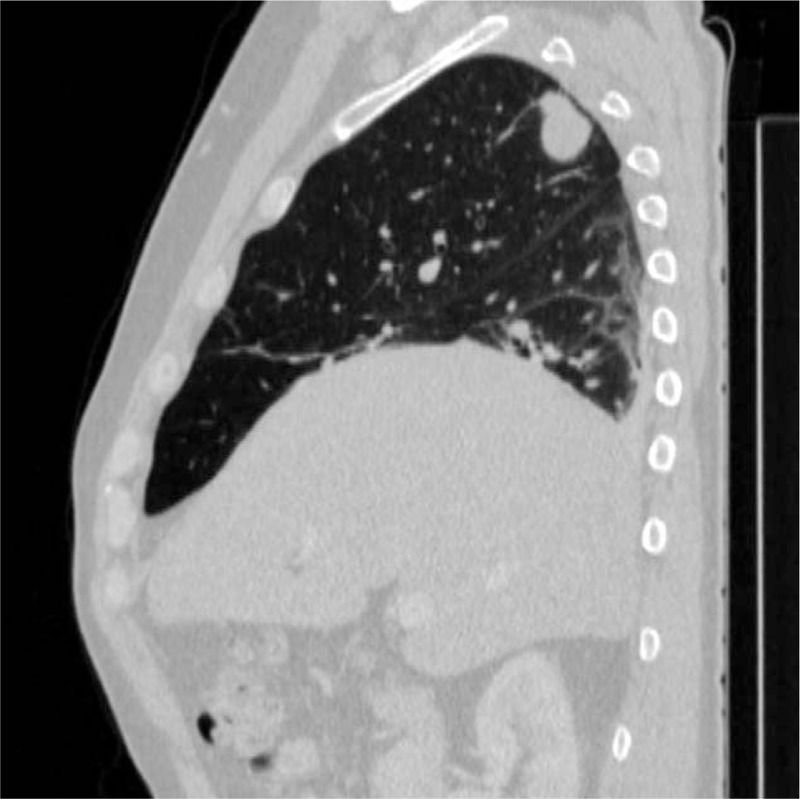
CT scan of the thorax showing the presence of a mass lesion located in the dorsal segment of the right upper pulmonary lobe – sagittal sections. CT = computed tomography.

The computed tomography (CT) of the thorax, abdomen, and pelvis revealed the presence of a 23/19 mm (transverse) and 20 mm (cranial–caudal) mass lesion, respectively, having speculated margins, contrast enhancement, and areas of necrosis at the level of the dorsal segment of the right upper pulmonary lobe. The mass presented several extensions to the pleura and determined pleural invasion (Figs. 5 and 6). In addition, multiple mediastinal adenopathy were noticed, and some of them presented central necrosis. No oncologic abnormalities were found at the examination of the abdomen and pelvis. Also, the cerebral CT scan did not show any pathological findings.

Laboratory findings were normal, except for the presence of inflammatory syndrome, slight anemia, and high values of vascular endothelial growth factor (VEGF) (1291 pg/mL, with normal values less than 100 pg/mL).

The patient was admitted to the Pneumology Clinic, where biopsy from the lateral cervical adenopathy was performed (tumor excision was not an option, due to the presence of multiple adenopathy). The anatomopathological examination of multiple fragments from the lymph node revealed multiple neoplastic infiltrates, suggestive of adenocarcinoma metastasis.

Positron emission tomography with 2-deoxy-2-[fluorine-18]fluoro-D-glucose confirmed the presence of the pulmonary malignant tumor with multiple active adenopathy.

Based on the clinical examination that revealed signs and symptoms suggestive of Pierre Marie–Bamberger syndrome, the X-ray examination that showed abnormalities compatible with the same pathology mentioned earlier, and on the positron emission tomography and histologic findings, the diagnosis of pulmonary adenocarcinoma with lymph nodes metastases and paraneoplastic hypertrophic osteoarthropathy was established.

The patient received treatment with nonsteroidal antiinflammatory drugs and opiate analgesics, which relieved the pain without any adverse event; he was then referred to the Oncology Department for further treatment of the primary pathology. The patient was treated with different types of chemotherapeutics, immunotherapy, and gamma-knife radiotherapy for cerebral metastases. Unfortunately, the disease progressed despite all of these therapeutic measures and the patient died 9 months later.

## 3 Discussion

Our patient presented to the Neurology Department for what was considered a neuropathic type of pain. However, it was to be noticed that the pain was not limited to a root, nerve, or plexus territory, it was not accompanied by objective sensory deficit, and it did not exacerbate with cervical spine movement, although it became worse when pressure was applied on bone extremities. All of these features helped us to establish that the patient presented nociceptive pain. Of note, the pain was associated with digital clubbing, progressive volume increase of all the 4 extremities, and cutaneous abnormalities. The digital clubbing, also termed Hippocratic fingers, could have been idiopathic (almost half of the cases) or secondary to other pathologies (i.e., pulmonary, cardiac, or gastric).

In this case, few clinical diagnostic hypotheses had to be tested. The first 2 possible causes were acromegaly or pseudoacromegaly; however, the patient had normal tongue volume, digital clubbing, and lateral cervical adenopathy. Another differential diagnosis was thyroid acropathy; however, seric levels of thyroid hormones were normal in our patient and he was never treated for such a disease. The most probable clinical diagnosis was Pierre Marie–Bamberger syndrome, as our patient presented most of its clinical features.

Pierre Marie–Bamberger syndrome is a rare osteocutaneous disease characterized by hypertrophy especially of the long bones and surrounding soft tissues due to a process of subperiostal bone proliferation that starts with the distal ends of these long bones. Secondary to proliferative periostitis and periosteal ossification, there is an increase in the circumference of the long bones, but it is worth mentioning that there is no increase in their length.^[5]^ These osseous abnormalities were also observed in the case of our patient when radiographic examinations were performed.

Periostosis is commonly seen in patients with hypertrophic osteoarthropathy. Digital clubbing of the upper and lower limbs is the most frequent manifestation of this syndrome; conversely, thickening of the skin is less common.^[6]^ All of these manifestations were present in our patient.

The diagnosis of hypertrophic osteoarthropathy is based on clinical symptoms and radiographic findings.^[7]^

In large series of cases, the prevalence of lung neoplasm in patients with hypertrophic osteoarthropathy varied between 4% and 32%.^[8]^ In a study that enrolled 81 patients with bronchopulmonary malignant tumors from our country, the authors found that 31.6% of the patients had a complete or incomplete Pierre Marie–Bamberger syndrome.^[9]^ It is considered that the prevalence is higher in patients with nonsmall-cell lung tumors than in those with small-cell lung carcinoma probably due to a longer survival and a better prognosis of the former.^[6]^ Among lung malignant tumors, hypertrophic osteoarthropathy occurs most frequently in patients with adenocarcinoma.^[10]^

Therefore, in our case, hypertrophic osteoarthropathy was considered as a paraneoplastic syndrome associated to lung adenocarcinoma.

Approximately 20% of patients with isolated digital clubbing are diagnosed with lung malignant tumors suggesting that such patients should be evaluated for pulmonary malignancy even in the absence of respiratory symptoms in order to start the treatment early and to ensure a better prognosis.^[10]^

Regarding the high VEGF levels found in case of our patient, there could be few explanations linked to the pathophysiology of this syndrome. One of them is that the tumor produces and expresses growth factors like VEGF, platelet-derived growth factor, growth hormone, and also gonadotropins.^[11]^ Another theory suggests that due to pulmonary shunting, platelet precursors cannot be fragmented at this level and pass into systemic circulation releasing growth factors, including VEGF, when they are trapped in the peripheral capillaries.^[1]^ Among other growth factors, VEGF plays an important role in the development of digital clubbing, periostosis, and effusion of small joints by promoting angiogenesis, vasodilatation, hyperplasia, interstitial edema, and collagen deposition (in tissues). Furthermore, VEGF has a direct effect on osteoblasts and osteoclasts.^[2]^ As stated by Armstrong et al,^[6]^ in lung carcinoma, there may be some degree of shunting, secondary to local tissue destruction; however, the main source of growth factors are the malignant cells, and this hypothesis is supported by the resolution of the clubbing after tumor resection in most of the patients.

In a retrospective study of 6151 lung cancer patients, 1.87% had signs and symptoms of hypertrophic osteoarthropathy, and the majority of cases consisted in male smoker patients having adenocarcinoma and advanced disease. The symptoms and imaging findings (bone scintigraphy) improved in patients who received treatment, especially in those who underwent tumor excision.^[12]^ Practically, the treatment and prognosis of Pierre Marie–Bamberger syndrome are related to the primary etiology. In the case of the patients in whom primary etiology cannot be treated, symptomatic treatment (unilateral vagotomy, adrenergic blockade, nonsteroidal antiinflammatory drugs, octreotide, bisphosphonates, and specific inhibitors, e.g., for epidermal growth factor receptor or VEGF) proved its benefits.^[13,14]^ Recent research showed that cisplatin and other chemotherapeutic agents like doxorubicin directly reduced VEGF transcription indicating a different mechanism of action of such drugs in hypertrophic osteoartropathy.^[15]^

This is the case of an overlooked pathology in a patient referred to the clinic for further investigations of a pain considered to be neuropathic, secondary to small cervical protrusions. The patient proved to present a nociceptive type of pain. Pierre Marie–Bamberger syndrome should be considered in this type of patients as it can be associated with numerous diseases especially neoplastic type.

## Author contributions

**Conceptualization:** Athena Cristina Ribigan, Ovidiu Alexandru Bajenaru.

**Data curation:** Athena Cristina Ribigan, Ovidiu Lucian Bajenaru, Florina Anca Antochi.

**Investigation:** Athena Cristina Ribigan, Ovidiu Lucian Bajenaru, Florina Anca Antochi, Ovidiu Alexandru Bajenaru.

**Supervision:** Ovidiu Alexandru Bajenaru.

**Writing – original draft:** Athena Cristina Ribigan.

**Writing – review & editing:** Athena Cristina Ribigan, Florina Anca Antochi, Ovidiu Alexandru Bajenaru.

## References

[1] Yap FY Skalski MR Patel DB Hypertrophic osteoarthropathy: clinical and imaging features. Radiographics 2017;37:157–95. 27935768 10.1148/rg.2017160052

[2] Chakraborty RK Sharma S Secondary Hypertrophic Osteoarthropathy. In: StatPearls. Treasure Island, FL, 2018. https://www.ncbi.nlm.nih.gov/books/NBK513342/ Accessed November 2018.

[3] Meyer HJ Leifels L Bach AG Secondary hypertrophic osteoarthropathy caused by non-pleural or pulmonary tumors. Medicine (Baltimore) 2017;96:e7985. 28885355 10.1097/MD.0000000000007985 PMC6392738

[4] Yao Q Altman RD Brahn E Periostitis and hypertrophic pulmonary osteoarthropathy: report of 2 cases and review of the literature. Semin Arthritis Rheum 2009;38:458–66. 18760449 10.1016/j.semarthrit.2008.07.001

[5] Cannavo SP Guarneri C Borgia F Pierre Marie-Bamberger syndrome (secondary hypertrophic osteoarthropathy). Int J Dermatol 2005;44:41–2. 10.1111/j.1365-4632.2004.02351.x 15663658

[6] Armstrong DJ McCausland EM Wright GD Hypertrophic pulmonary osteoarthropathy (HPOA) (Pierre Marie-Bamberger syndrome): two cases presenting as acute inflammatory arthritis. Description and review of the literature. Rheumatol Int 2007;27:399–402. 17006703 10.1007/s00296-006-0224-2

[7] Ito T Goto K Yoh K Hypertrophic pulmonary osteoarthropathy as a paraneoplastic manifestation of lung cancer. J Thorac Oncol 2010;5:976–80. 20453688 10.1097/JTO.0b013e3181dc1f3c

[8] Boyer-Duck E Dajer-Fadel WL Hernandez-Arenas LA Pierre-Marie-Bamberger syndrome and solitary fibrous tumor: a rare association. Asian Cardiovasc Thorac Ann 2018;26:154–7. 29378437 10.1177/0218492318757042

[9] Suteanu S Rohan C Gherasim E Hypertrophic osteoarthropathy secondary to bronchopulmonary cancer (our experience). Rom J Intern Med 1992;30:281–4. 1299419

[10] Poanta L Parasca I Fazakas E Paraneoplastic hypertrophic osteoarthropathy: evaluation at 25 years after pneumectomy. Pol Arch Med Wewn 2009;119:603–6. 19776708

[11] Pourmorteza M Baumrucker SJ Al-Sheyyab A Hypertrophic pulmonary osteoarthropathy: a rare but treatable condition in palliative medicine. J Pain Symptom Manage 2015;50:263–7. 25701054 10.1016/j.jpainsymman.2015.02.005

[12] Qian X Qin J Hypertrophic pulmonary osteoarthropathy with primary lung cancer. Oncol Lett 2014;7:2079–82. 24932292 10.3892/ol.2014.2022 PMC4049689

[13] Chakraborty RK Sharma S Secondary Hypertrophic Osteoarthropathy. In: StatPearls. Treasure Island, FL, 2019. https://www.ncbi.nlm.nih.gov/books/NBK513342/ Accessed April 2019.

[14] Kilaru M Vitale C Montagnini M Pain management in hypertrophic pulmonary osteoarthropathy: an illustrative case and review. Am J Hosp Palliat Care 2012;29:302–7. 21998443 10.1177/1049909111421608

[15] Openshaw MR Rowan CS Grumett S Atypical hypertrophic osteoartropathy as a presenting complaint in a non-smoker. J Med Cases 2013;4:204–7.

